# Non-traumatic Ocular Findings in Industrial Technical Workers in Delta State, Nigeria

**DOI:** 10.4103/0974-9233.48864

**Published:** 2009

**Authors:** A. E. Omoti, O. T. Edema, F. B. Akinsola, P. Aigbotsua

**Affiliations:** 1 From the Department of Ophthalmology, University of Benin Teaching Hospital, Nigeria; 2 From the Department of Surgery, College of Medicine, University of Lagos, Nigeria; 3 From the Department of central Hospital, Warri, Delta State, Nigeria

**Keywords:** Non-ocular Trauma, Disorders, Industrial, Workers

## Abstract

**Purpose:**

To determine the pattern of non-traumatic ocular disorders in industrial technical workers in the Delta state, Nigeria.

**Methods:**

A cross-sectional study of the pattern of non-traumatic ocular disorders among industrial technical workers in 3 factories in Ughelli North local Government Area of Delta state, Nigeria was conducted between February, 2002 and May, 2002. In addition to the demographic, the workers were studied for the presence of any non-trauma related ocular findings. Visual acuity of these workers was obtained as well as ocular examination was performed by using the Snellen's chart, pen torch, ophthalmoscope, Perkins hand-held tonometer, Ishihara plates. Patients were refracted if their visual acuity was less than normal.

**Results:**

Five hundred technical workers were screened that included 200 (40%) from the construction industry, 180 (36%) from the rubber factory and 120(24%) from the oil mill. All the workers studied were males. Ocular disorders were seen in 664 (66.4%) of the eyes. The most common ocular disorders were pingueculum 215 (21.5%), presbyopia 97 (9.7%), refractive error 94 (9.4%), pterygium 86 (8.6%) and chronic conjunctivitis 45 (4.5%). None of the workers was blind from non-traumatic causes. Only 36 (7.2%) workers wore any protective eye devices at work.

**Conclusion:**

Non-traumatic ocular disorders are common in the industrialized technical workers in the Delta state of Nigeria. The use of protective eye devices is low in these workers and suggests that measures to implement ocular safety should be undertaken in these industries.

Presence of ocular disorders in industrial workers may result in visual impairment, suffering, reduced manpower, man hours and monetary loss. Most of these hazards are preventable if adequate precautionary measures are taken.^1^–^4^ Worker's eyes may be exposed to a variety of dangerous agents depending on the type of industry which may constitute an occupational hazard.^5^,^6^ Most of the reported studies related to non-traumatic ocular disorders have emerged from developed countries with relatively little information from developing countries such as Nigeria. For example, a study carried out at Wolver-Hampton, a highly industrialized area of the United Kingdom showed 73.8% of all ocular trauma over a 10 year period occurred in industries.^5^ A similarly higher figure of 71% was reported in an early study conducted in 1923.^6^ On the other hand, a much lower figure of 15.4% was reported from a relatively less industrialized area of Northern Ireland.^7^

Some of the sources of industrial ocular hazards may include exposure to dangerous agents. For example, acute exposure to ultra-violet radiation results in photokeratitis characterized by pain and grittiness that may cause decreased corneal sensitivity and damage to the corneal endothelium.^8^,^9^ Long term exposure may be partly responsible for conditions such as pterygia, pingueculae, band-shaped keratopathy and climatic droplet keratopathy.^10^–^12^ Repeated exposure to radiant energy on glass blowers, steel workers, blast furnace attendants and blacksmiths can result in glassblower cataract.^3^ The damage to the lens is a consequence of absorption of direct and indirect infrared radiation.^13^,^14^

The aim of this study is to identify the pattern of non-traumatic ocular disorders in industrial technical workers in some of the industries in Ughelli North local government area of Delta state, Nigeria.

## MATERIALS AND METHODS

A cross-sectional survey of industrial workers in Ughelli North Local Government Area (LGA) of Delta state of Nigeria was conducted between February, 2002 and May 2002. Delta state is a major oil producing state located in the Niger Delta region of Nigeria. Three of the 5 major industries in Ughelli North LGA participated in the study which included Peanard Oil (OIL MILL), Imoniyame Rubber Crump factory (RUBBER FAC) and ANCOG Nigeria Limited (CONSTR), a construction and civil engineering industry that also possesses an asphalt plant. Only the technical workers of these three industries were studied. Administrative and other non-technical workers were excluded from the study in all the industries. After screening, 500 workers of various occupations were found to be eligible to participate in the study. Informed consent was obtained from all the workers included in the study. The screened workers had various occupations including electricians, mechanics, machine operators, welders, furnace operators, rubber washers, driers, asphalt pavers, cake packers and tailors.

Ethical committee approval was obtained from the University of Benin Teaching Hospital (UBTH). Confidentiality was guaranteed and workers who expressed a willingness to withdraw from the study were given the right to do so. The authors administered the questionnaires that was prepared for this study and examined the workers. All interviews and examination conducted during the study took place within the factory site or out-doors. The use of proper eye care protection was emphasized at the end of screening sessions. Demographic of these patients including personal data, industrial work history, ocular history and examination findings were recorded.

Visual acuity was measured using the Snellens's literate chart for distance placed at six meters either outdoors in daytime or inside the examination room with adequate illumination. Each eye was tested separately with and without glasses where applicable and subsequently refracted. Near visual acuity was tested using Jaegar's reading chart.

Color vision was tested using the Ishihara pseudo-isochromatic plates and findings noted as simply normal or abnormal. External ocular examination was carried out with a pen-torch and loupe with × 4 magnification. Fluorescein staining of corneal lesions was done whenever indicated.

Further clinical assessment included the use of Keeler Vista 20 direct ophthalmoscope for fundoscopy. Dilated fundoscopy was achieved with 2.5% phenylephrine hydrochloride and 0.5% mydriaticum for individuals with clear cornea but impaired red reflex, small pupils or severely reduced vision <6/60, excluding those with shallow anterior chamber. Tonometry was done using Perkin's hand-held applanation tonometer after the instillation of xylocaine eye drop and florescein dye. Visual field examination was assessed by the Bjerrum's screen when there was elevated intraocular pressure and/or when the optic discs were suspicious or pathologically cupped. Subjects were informed about the findings and those that required any treatment were properly treated or referred to the Federal Medical Centre, Asaba for further treatment.

## RESULTS

A total of 500 industrial technical workers were screened that included 200(40%) from the construction industry, 180 (36%) from the rubber factory, and 120 (24%) from the oil mill. All the workers were males. The mean age of these workers was 33.61 years (SD± 11.1). The age distribution of the workers is shown in Table 1. The distribution of non-traumatic ocular disorders in these workers is shown in Table 2. All together, 354 workers (70.8%) had ocular disorders. Of the 664 eyes affected, the most common ocular disorders were pingueculum (21.5%), presbyopia (9.7%), refractive error (9.4%) and pterygium (8.6%). The distribution of visual acuity in the workers is shown in Table 3. None of the workers was blind from non-traumatic causes. However, 13 workers had monocular blindness from traumatic causes including 10 from the construction firm and 3 from the oil mill.

**Table 1:** Age Distribution of Industrial Workers Studied.

		Factories				
Age Group (Yrs)	Construc.	Rubber Fac.	Oil Mill	Total No	%
20	8 (32.0%)	5 (48.0%)	12 (48.0%)	25	5.0

21-30	70 (31.0%)	66 (29.2%)	90 (39.8%)	226	45.2

31-40	51 (41.5%)	66 (52.6%)	6 (4.9%)	123	24.6

41-50	34 (52.3%)	25 (38.5%)	6 (9.2%)	65	13.0

51-60	32 (59.2%)	16 (29.6%)	6 (11.0%)	54	10.8

>60	5 (71.0%)	2 (28.6%)	-	7	1.4

Total	200	180	120	500	100

**Table 2:** Distribution of Ocular Disorders in the Three Factories Studied.

			Facories		
Ocular Disorders	Construc. (Of 400 Eyes)	Rubber Fac. (Of 360 Eyes)	Oil Mill (Of 240 Eyes)	Total Eyes (1000)	%
Pingueculum	90 (22.5%)	98 (27.3%)	27 (11.2%)	215	21.5

Presbyopia	41 (10.3%)	42 (11.6%)	14 (5.8%)	97	9.7

Refractive error	39 (9.7%)	42 (11.6%)	13 (5.4%)	94	9.4

Pterygium	39 (9.7%)	44 (12.2%)	3 (1.2%)	86	8.6

Choronic conjunctivitis	34 (8.5%)	4 (1.1%)	7 (2.9%)	45	4.5

Optic neuropathy	22 (5.5%)	5 (1.4%)	-	27	2.7

Glaucoma	10 (2.5%)	9 (2.5%)	7 (2.9%)	26	2.6

Conjunctival naevus	7 (1.7%)	9 (2.5%)	10 (4.2%)	26	2.6

Macula disease	7 (1.7%)	7 (1.9%)	-	14	1.4

Hypertensive retinopathy	4 (1.0%)	4 (1.1%)	-	8	0.8

Retinal detachment	2 (0.5%)	-	6 (2.5%)	8	0.8

Cataract	5 (1.2%)	-	-	5	0.5

Corneal opacity	2 (0.5%)	-	3 (1.3%)	5	0.5

Chorioretinal scar	2 (0.5%)	2 (0.5%)	-	4	0.4

Optic atrophy	2 (0.5%)	-	-	2	0.2

Chalazion	-	2 (0.5%)	-	2	0.2

Total	306 (76.5%)	268 (74.4%)	90 (37.5%)	664	66.4

**Table 3:** Distribution of Visual Acuity in Better Eye of Industrial Workers.

Factory		Visual Acuity		
	6/4 – 6/18	6/18-6/36	<6/36	Total
Construc.	182 (91.0%)	15 (7.5%)	3 (1.5%)	200
Rubber Fac.	173 (96.1%)	7 (3.9%)	-	180
Oil Mill	116 (96.7%)	-	4 (3.3%)	120
Total	471 (94.2%)	22 (4.4%)	7 (1.4%)	500

Sand dust, chemicals and cake dust were the most common harmful agents. Only 36 workers (7.2%) were wearing protective eye devices during work in the 3 industries surveyed. These include 22 (11%) in the construction firm, 11(6%) in the rubber factory and 3 workers (2.5%) in the oil mill.

## DISCUSSION

The pattern of ocular disorders found in our study is relatively similar to the finding in similar studies in Nigeria.^15^,^16^ However there are differences in the order of occurrence of these disorders. The most common disorders in this study in descending order were pingueculum, presbyopia, pterygia and refractive error. In Kaduna (Northern Nigeria), conjunctivitis, corneal opacity, pingueculum and pterygium were the most commom.^15^ In Enugu (Eastern Nigeria),^16^ presbyopia, refractive error, pterygium and cataract were the most common. These differences may be related to the differences in the types of industries studied and in the climatic conditions in the different cities studied. The majority of workers in this study were out-door workers. This may be associated with chronic exposure to ultraviolet radiation resulting in pingueculum and pterygium. Thus, it is not surprising that pingueculum was the most common ocular disorder in this study. As expected, refractive errors and presbyopia were high on the list. This may be related to the common occurrence of these conditions in the general population.^19^,^20^

To our knowledge, only few similar studies in industrial technical workers have been carried out in urban centers in Nigeria.^15^–^18^ Results from study showed that the vast majority of workers from the three industries surveyed, had some kind of ocular disorders. These findings are in agreement with previously reported studies in which over 66% industrial workers had some kind of ocular disorders. Ocular disorders from our study were higher than the finding by Abiose and Otache^15^ who reported that 397 of the 3,676 industrial workers (10.8%) examined had ocular disorders. However, in their study both the technical and non-technical workers were included. Okoye,^16^ on the other hand reported that 528 out of 646 industrial technical workers (81.7%) had ocular disorders which is higher than the finding from our study. This difference may be due to differences in the type of industries studied as they included cement, coal, vehicle repair, and timber factories.

The harmful agents to which the industrial workers are exposed may vary according to the type of industry studied. The agents used in the various factories may determine the type of ocular disorder that may be caused by the harmful agents. In this report, sand dust, chemicals and cake dust were the most common harmful agents. These are the substances emitted and used in the industries studied in this report. In contrast to this, Okoye^16^ in Eastern Nigeria reported that the most common harmful agents were metal chips, cement dust, coal stone, wood fragments and welder's arc rays. This is because the industries he studied were cement, coal, vehicle repair and timber factories.

The overwhelming majority of workers in this study did not wear protective eye devices which is similar to the finding from a similar study from Nigeria.^16^ This is because of lack of awareness and implementation of safety regulation by the management of these industries. An even higher figure of 96.4% was reported among floor workers in North-India.^21^ The North Indian town studied was underdeveloped, and the reason for neglect of the use of protective glasses was similar to ours.

In conclusion, non-traumatic ocular disorders were common in industrial technical workers in the Delta region of Nigeria. These workers were usually unaware of or not provided with protective eye devices. It is recommended that there should be enforcement of legislation requiring these industries to provide their workers eye safety protective wear while at work. Further, workers should be educated regarding the use of eye protection and better eye care services be provided for those who sustain ocular disorders while on the job in these industries.
